# Genetic Variants and Their Interactions in the Prediction of Increased Pre-Clinical Carotid Atherosclerosis: The Cardiovascular Risk in Young Finns Study

**DOI:** 10.1371/journal.pgen.1001146

**Published:** 2010-09-30

**Authors:** Sebastian Okser, Terho Lehtimäki, Laura L. Elo, Nina Mononen, Nina Peltonen, Mika Kähönen, Markus Juonala, Yue-Mei Fan, Jussi A. Hernesniemi, Tomi Laitinen, Leo-Pekka Lyytikäinen, Riikka Rontu, Carita Eklund, Nina Hutri-Kähönen, Leena Taittonen, Mikko Hurme, Jorma S. A. Viikari, Olli T. Raitakari, Tero Aittokallio

**Affiliations:** 1 Biomathematics Research Group, Department of Mathematics, University of Turku, Turku, Finland; 2 Department of Clinical Chemistry, Tampere University Hospital and University of Tampere, Tampere, Finland; 3 Data Mining and Modeling Group, Turku Centre for Biotechnology, Turku, Finland; 4 Department of Clinical Physiology, Tampere University Hospital and University of Tampere, Tampere, Finland; 5 Department of Medicine, Turku University Central Hospital, Turku, Finland; 6 Research Centre of Applied and Preventive Cardiovascular Medicine, University of Turku, Turku, Finland; 7 Department of Clinical Physiology and Nuclear Medicine, Kuopio University Hospital and University of Eastern Finland, Kuopio, Finland; 8 Department of Microbiology and Immunology, University of Tampere, Tampere, Finland; 9 Department of Pediatrics, Tampere University Hospital, Tampere, Finland; 10 Department of Pediatrics, University of Oulu, Oulu, Finland; 11 Department of Medicine, University of Turku, Turku, Finland; 12 Department of Clinical Physiology, Turku University Hospital, Turku, Finland; University of California San Diego and The Scripps Research Institute, United States of America

## Abstract

The relative contribution of genetic risk factors to the progression of subclinical atherosclerosis is poorly understood. It is likely that multiple variants are implicated in the development of atherosclerosis, but the subtle genotypic and phenotypic differences are beyond the reach of the conventional case-control designs and the statistical significance testing procedures being used in most association studies. Our objective here was to investigate whether an alternative approach—in which common disorders are treated as quantitative phenotypes that are continuously distributed over a population—can reveal predictive insights into the early atherosclerosis, as assessed using ultrasound imaging-based quantitative measurement of carotid artery intima-media thickness (IMT). Using our population-based follow-up study of atherosclerosis precursors as a basis for sampling subjects with gradually increasing IMT levels, we searched for such subsets of genetic variants and their interactions that are the most predictive of the various risk classes, rather than using exclusively those variants meeting a stringent level of statistical significance. The area under the receiver operating characteristic curve (AUC) was used to evaluate the predictive value of the variants, and cross-validation was used to assess how well the predictive models will generalize to other subsets of subjects. By means of our predictive modeling framework with machine learning-based SNP selection, we could improve the prediction of the extreme classes of atherosclerosis risk and progression over a 6-year period (average AUC 0.844 and 0.761), compared to that of using conventional cardiovascular risk factors alone (average AUC 0.741 and 0.629), or when combined with the statistically significant variants (average AUC 0.762 and 0.651). The predictive accuracy remained relatively high in an independent validation set of subjects (average decrease of 0.043). These results demonstrate that the modeling framework can utilize the “gray zone” of genetic variation in the classification of subjects with different degrees of risk of developing atherosclerosis.

## Author Summary

Although cardiovascular events, such as myocardial infarction and stroke, usually occur at later ages, it is known that the atherogenic process begins much earlier in life. Detection of subclinical atherosclerosis would therefore offer the means to identify individuals who are at increased risk of developing cardiovascular events. What remains unclear is the relative contribution of genetic variation to the development of the early stages of atherosclerosis. To address this question, we searched for combinations of both genetic and clinical determinants that are the most predictive of the progression of subclinical carotid atherosclerosis in a sample of 1,027 young adults, aged between 24–39 years, from the Finnish general population (The Cardiovascular Risk in Young Finns Study). We demonstrate here, for the first time in a population-based follow-up study, a predictive relationship between individual's genotypic variation and early signs of atherosclerosis, which cannot be explained by conventional cardiovascular risk factors, such as obesity and elevated blood pressure levels. The predictive modeling framework facilitates the usability of genetic information by identifying informative panels of variants, along with conventional risk factors, which may prove to be useful in early detection and management of atherosclerosis. The clinical implications of these findings remain to be studied.

## Introduction

A major challenge of medical genetics is to determine an optimal set of genetic markers, typically in the form of single nucleotide polymorphisms (SNP), which when combined together with conventional risk factors, could be used in individual level risk prediction, classification and clinical decision-making. However, genome-wide association studies (GWAS) have demonstrated that the ubiquitous heritability of most common disorders is due to multiple SNPs of small effect size and even an aggregate of these effects is not yet predictive enough for clinical utility [1]. It has therefore been suggested that the traditional case-control studies, which focus on qualitative phenotypes such as diagnosed cases versus controls, could be complemented by population-based cohort studies, which profile quantitative clinical phenotypes and how they change over time in individuals who are representative of the general population. Consequently, certain common disorders may be interpreted as being the extremes of the quantitative phenotypes that are continuously distributed over the population [1]. Comparing various ranges of the low and high extremes of such quantitative traits, rather than dichotomizing the same distribution exclusively into cases and controls, can offer the means to increase the statistical power of the variants [2]–[5], uncover molecular pathways and networks behind various subtypes and progression stages [6], and eventually even help to improve the early diagnosis, treatment and prevention of the most extreme cases. The objective here was to systematically investigate the potential of this extreme selection strategy to provide predictive insights into the early development of atherosclerosis, using the carotid IMT as a quantitative phenotype and our unique population-based follow-up study of atherosclerosis precursors as a basis for sub-sampling of subjects with increasing disease risk.

Atherosclerosis is a common disorder which develops due to the complex interplay of various genetic and environmental factors, most of which are still poorly understood. It is known that conventional cardiovascular risk factors, such as obesity, elevated blood pressure and high low-density lipoprotein (LDL) cholesterol levels, play an important role in the risk of its progression into severe clinical manifestations, for instance, coronary heart disease (CHD) [7], [8]. Recently, a number of genetic risk markers that associate with coronary disease outcomes and serum lipid concentrations have also been identified in case-control settings [9]–[21]. However, the relative contribution of genetic variation to the early stages of the cardiovascular disease remains unclear. From the experimental design point of view, the subtle inter-individual phenotypic variability makes it difficult to prognosticate clear-cut cases and controls in a pre-clinical setting, thereby limiting the capability of the cross-sectional case-control designs in distinguishing the variants associated with an increased progression risk from the background variability. An additional challenge is that even in the absence of significant single-marker effects, multiple genetic markers from distinct molecular pathways may act synergistically when combined, leading to different atherosclerosis phenotypes. Confounding inter-individual variation and interactions across the genetic and conventional risk factors can also mask the phenotypic variation, especially when studying composite phenotypes such as LDL-cholesterol levels [22]. Therefore, a well-defined quantitative measurement that reflects the full spectrum of the disease progression is needed, together with an efficient computational approach, to systematically explore the genotype-phenotype relationships across different development stages of atherosclerosis.

Measurement of the carotid artery intima-media thickness (IMT) is an established, intermediate phenotype of atherosclerosis that has been used, for instance, to investigate the development of pre-clinical atherosclerosis [23], [24], and to predict the onset of future cardiovascular events, such as myocardial infraction and stroke [25]–[27]. It can be measured non-invasively through the use of ultrasound imaging in large populations of healthy subjects, without the biases related to clinically diagnosed cases and controls [28], making it an ideal quantitative measurement for stratifying subjects into various risk classes. However, comparisons of such risk classes using statistical significance testing procedures that consider only one SNP at a time may yield sub-optimal findings when exploring the genotype-specific effects of large number of SNPs, given that these modest phenotypic effects are likely to be characterized by substantial genetic heterogeneity among multiple variants [29]–[31]. Accordingly, it has been argued that the statistics being used to identify variants that are significantly associated with the disease risk - typically odds ratios or *p*-values for association - are not the most appropriate means for evaluating the predictive or clinical value of the genetic profiles [32], [33]. For example, the individual SNPs with the strongest statistical support in coronary artery disease-related case-control studies seem to have only a minor, if any, role in predicting carotid IMT or its progression, when compared to the conventional risk factors [34], [35]. In fact, these susceptibility variants are able to provide only a marginal and inconsistent improvement even in the discrimination of the CHD cases or prediction of cardiovascular events [36]–[41], thus hindering the value of these ‘top hits’ for diagnostic prediction. Moreover, additional challenges stem from the identification of gene-gene and gene-environment interactions, which are thought to be profoundly important in the development of many complex diseases [29], [30], [42].

In the present analysis from the Young Finns Study, we took a more holistic approach towards revealing the contribution of genetic variation to the early progression of atherosclerosis. The approach was based on a stratified sampling and comparison of the increasing risk classes from our longitudinal population cohort. Rather than using the conventional single-SNP statistical significance testing in the identification of risk-modifying variants and their interactions, we explicitly searched for those subsets of SNPs that are the most predictive of the increasing risk classes by means of a predictive modeling framework using a machine learning-based SNP-subset selection procedure. The predictive approach was used here to mine those associations that did not necessary meet the stringent levels of statistical significance at the level of individual SNPs, yet still having significant contribution to the combined predictive power at the level of SNP-subsets. In particular, we addressed the following questions: (*i*) whether the genetic variants can improve the prediction accuracy of IMT-based risk classes beyond that obtained with conventional risk factors; (*ii*) which variants are the most predictive of the subjects that show extreme IMT levels either at the baseline or in the follow-up study, or progression over the 6-year period; (*iii*) whether the predictive SNP-panels also include other variants than those risk markers identified in the previous case-control association studies; and (*iv*) whether the machine learning-based SNP selection can provide variants with increased predictive power compared to the SNPs with the greatest statistical significance in the present study population. We also illustrate how the predictive modeling framework can be employed to identify epistasis interactions among genetic variants that are related to the disease progression. Finally, as the first step toward elucidating functional mechanisms behind the genetic variants and their interactions, we also mapped the biological pathways and processes that underlie those variants most predictive of the extreme progression cases.

## Results

**Figure 1:**
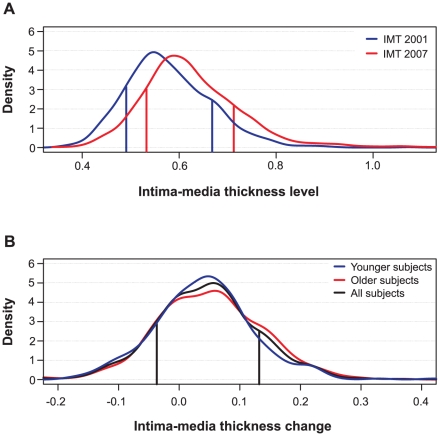
Distributions of intima-media thickness (IMT) of the study subjects. (A) IMT levels in the baseline and follow-up studies in 2001 and 2007, respectively. (B) IMT changes from 2001 to 2007. The age-stratified distributions depict the baseline age groups of 24–30 and 33–39 years (Younger and Older subjects), as well as their combined distribution (All subjects). The vertical lines indicate the representative 15% and 85% quantile points (*q*) that divide the subjects into two risk groups: the low-risk class (subjects with the lowest *q*% of IMT levels or changes) and the high-risk class (subjects with the highest *q*% of IMT levels or changes).

The baseline study cohort in 2001 was comprised of 1,027 subjects from the Finnish general population, aged 24–39 years, with complete data including both the ultrasound-based imaging of the carotid IMT and the blood sample-based genotyping of the candidate SNPs (see Table S1); of these subjects, 813 also participated in the 2007 follow-up study of the IMT progression (see Materials and Methods for details). The relative contribution of the SNPs to the individual IMT levels was evaluated by means of a predictive modeling framework, in which the study subjects were first divided into gradually increasing low-risk and high-risk classes according to the quantile points, say (1-*q*) and *q*, of their pooled IMT distribution (*q* ranges from 5% to 25%; see Figure 1). A non-linear Bayesian classifier was implemented here as the predictive model (see Materials and Methods for details). Using both the genetic and conventional risk factors collected in the baseline study in 2001 as predictor variables, we determined the most predictive risk factor combinations separately for both the 2001 and 2007 IMT risk classes, as well as the IMT progression between 2001 and 2007. For a comparison, the most significant genetic variants were determined using single-SNP statistical testing for the same risk classes. The area under the receiver operating characteristic curve (AUC), with cross-validation, was used to evaluate the predictive value of the different factor combinations, followed by independent validation set-based assessment of how well the predictive models can generalize to independent sets of subjects.

### Clinical characteristics of the study subjects

The quantitative distributions of the levels of IMT and its progression over the 6-year period are shown in Figure 1. The IMT levels in the study population showed a slightly positive-skewed enrichment of subjects with higher IMT values indicating an increased risk of atherosclerosis (Figure 1A). There was a significant difference in the IMT distributions between the 2001 and 2007 follow-up studies (Kolmogorov-Smirnov *D* = 0.234, *p*<0.001). As expected, the majority of the conventional risk factors measured in 2001, including age, sex and BMI, were strongly correlated with the IMT levels both in the 2001 and 2007 studies (Table 1). However, only two risk factors, waist circumference and apolipoprotein B (ApoB), correlated with the IMT progression (the 2007-2001 change). In particular, even if the age was the most significant correlate of the IMT levels in 2001 and 2007, its linear explanatory power turned out to be insignificant for the IMT progression. Accordingly, the distributions of the IMT progression were similar in the groups of younger and older subjects (*D* = 0.0791, *p>*0.10; Figure 1B). To keep the non-linear prediction problem as general as possible, the age-groups and sexes were pooled into a single continuous distribution; however, all the predictive models were adjusted for the baseline conventional risk factors (Table 1). This enabled us to examine, for instance, the added contribution of genetic variation to the IMT progression not explained by the variation in the conventional cardiovascular risk factors.

**Table 1:** The baseline characteristics in 2001 along with their correlations with the 2007 level and progression of intima-media thickness (IMT).

Conventional Risk Factor*	Mean (SD)	IMT 2001		IMT 2007		IMT Progression	
		*r* †	*p* ‡	*r* †	*p* ‡	*r* †	*p* ‡
Sex (% women)	55.3	0.132	<0.001	0.195	<0.001	0.086	NS
Age in 2001 (years)	31.7 (4.92)	0.290	<0.001	0.301	<0.001	0.041	NS
BMI (kg/m^2^)	25.2 (4.38)	0.152	<0.001	0.188	<0.001	0.094	NS
Waist circumference (mm)	84.0 (12.0)	0.189	<0.001	0.260	<0.001	0.133	0.006
Systolic blood pressure (mmHg)	117 (13.2)	0.180	<0.001	0.158	<0.001	0.044	NS
Diastolic blood pressure (mmHg)	70.6 (10.5)	0.220	<0.001	0.160	<0.001	−0.020	NS
Total cholesterol (mmol/L)	5.17 (0.99)	0.113	0.011	0.155	<0.001	0.082	NS
LDL cholesterol (mmol/L)	3.28 (0.86)	0.126	0.002	0.166	<0.001	0.087	NS
HDL cholesterol (mmol/L)	1.29 (0.32)	−0.037	NS	−0.107	NS	−0.089	NS
Triglycerides (mmol/L)	1.35 (0.86)	0.047	NS	0.131	0.007	0.099	NS
ApoA1 (g/L)	1.49 (0.26)	−0.052	NS	−0.085	NS	−0.039	NS
ApoB (g/L)	1.06 (0.27)	0.110	0.016	0.195	<0.001	0.138	0.003
Smoking (% subjects)	22.8	0.049	NS	0.007	NS	−0.011	NS

*The characteristics in 2001 were used as potential confounding risk factors in predictive models.

†Pearson correlation coefficient (*r*-value) was calculated using the risk factors collected in 2001.

‡Statistical significance (Bonferroni corrected *p*-value) is from the *t*-distribution with *n-2* df (*n* = 1,027 in 2001 and *n* = 813 in 2007); NS, non-significant.

### Prediction of baseline IMT using genetic variants

**Figure 2:**
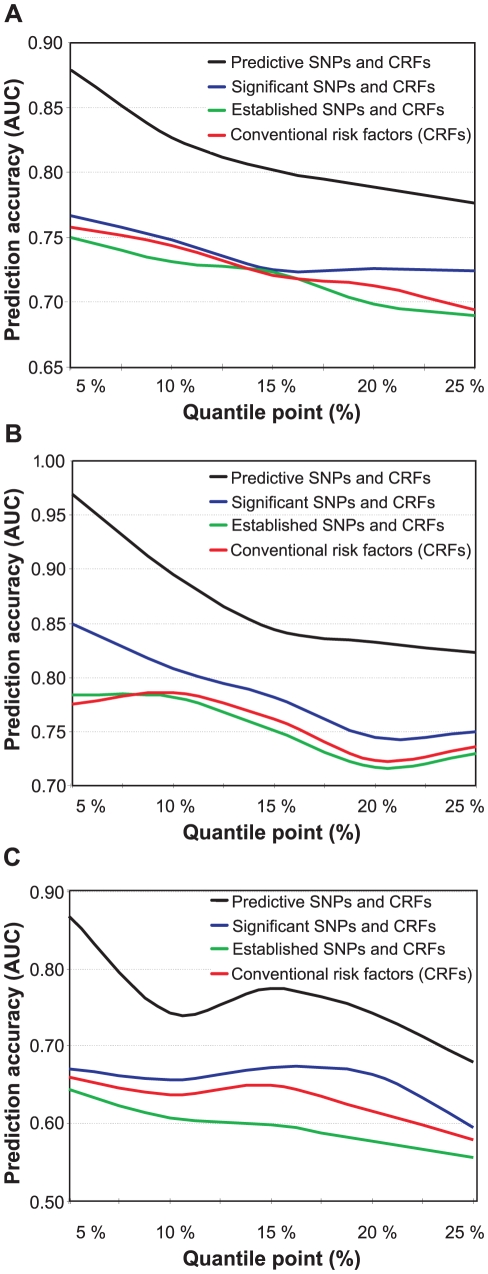
Prediction accuracy as a function of increasing risk classes. The accuracy was defined using the area under the receiver operating characteristic curve (AUC), and the risk classes using the quantile points (5–25%). (A) Prediction of the baseline IMT risk classes in 2001 when using the conventional risk factors either alone, or when combined with the panel of 17 SNPs associated in previous studies with cardiovascular morbidity (Established SNPs), with those SNPs that are significantly associated with the low- and high-risk classes (Significant SNPs), or with the most predictive SNPs identified using the machine learning-based approach (Predictive SNPs). (B) Prediction of the follow-up IMT risk classes in 2007 using the baseline conventional and genetic risk factors measured in 2001. (C) Prediction of the IMT progression risk classes when using the baseline conventional and genetic risk factors measured in 2001 (the same as in (A,B)).

To assess whether the genetic variants can increase the prediction accuracy of the risk classes beyond that obtained with the conventional risk factors alone, we used the predictive modeling framework with a machine learning-based SNP selection. The predictive risk factor combinations selected using this procedure were able to significantly improve the prediction of the subjects across the spectrum of low-risk and high-risk classes in 2001 (Figure 2A), when compared to using the conventional risk factors (CRFs) either alone or combined with those SNPs that were significantly associated with the low- and high-risk differences in the study subjects (the significances of the SNPs are detailed in Table S2). Interestingly, the panel of genetic risk markers established in the previous case-control association studies alone had a predictive power similar to that of a random classifier (average AUC 0.489), and these SNPs could not improve the prediction of the IMT risk classes over and above of the conventional risk factors (Established SNPs and CRFs; Figure 2). As expected, the predictive accuracy gradually decreased when moving from 5% to 25% quantile level, as the risk classes became phenotypically more heterogeneous in terms of the quantitative IMT-levels (see Figure 1A). The variants most predictive of the subjects with 15% of the lowest and highest IMT-levels in 2001 are listed in Table 2, together with their gene annotation information and the single-SNP statistical and predictive powers.

**Table 2:** The single nucleotide polymorphisms (SNPs) predictive of the subjects with 15% lowest and highest IMT levels in 2001.

SNP ID* (rs number)	Gene symbol (HGNC name)	SNP location (Chr region)	Significance† (*p*-value)	Predictive power‡ (%AUC)
rs2073658	USF1	1q23.3	0.70	11.8
rs1205	CRP	1q23.2	0.02	10.6
rs805305	DDAH2	6p21.33	0.38	9.68
**rs3890182**	ABCA1	9q31.1	0.81	7.53
rs6929137	C6orf97	6q25.1	0.10	7.53
rs4073307	IGSF1	Xq26.1	0.71	6.45
**rs693**	APOB	2p24.1	0.53	6.45
rs3130340	INTERGENIC	6p21.32	0.11	6.45
rs599839	PSRC1	1p13.3	0.10	6.45
**rs754523**	INTERGENIC	2p24.1	1.00	5.38
rs1143634	IL1B	2q13	0.51	5.38
rs4404254	ICOS	2q33.2	0.16	4.30
rs2548861	WWOX	16q23.1	0.14	4.30
rs2553268	WRN	8p12	0.15	3.23
rs4937100	IL18	11q23.1	0.22	2.15
rs2516839	USF1	1q23.3	0.13	2.15

*The SNPs identified also in the previous case-control association studies [9]–[21] are boldfaced.

†The corrected *p*-values larger than one were truncated to unity.

‡The SNPs are arranged according to their contribution to the overall prediction accuracy (AUC).

### Prediction of follow-up IMT using genetic variants

The predictive power of the genetic variants that were selected using the machine learning-based procedure increased further when predicting the risk classes in the 2007 follow-up, even if the genetic and conventional risk factors collected in only the baseline study were used as predictors (Figure 2B). This result can partly be attributed to the progression of the disease condition over the six years in a part of the study subjects (see Figure 1A). In particular, the classes of the most extreme levels of the IMT could be predicted with reasonably high accuracy also using single-SNP statistical testing, whereas the panel of established SNPs either with or without the conventional risk factors again showed much poorer performance (Figure 2B). These results suggest that the genetic variants, especially those that were identified using the machine learning-based SNP selection (see Table 3), can encode significant information according to which it is possible to predict subjects who will belong to different risk classes later in their lives with accuracies beyond that obtained with the conventional risk factors. We note that the baseline 2001 IMT-level was not used in the reported results when predicting the 2007 risk classes; however, in the case when the baseline IMT-level was used as an additional predictor, the prediction accuracies became very close to perfect discrimination (AUC ranged from 0.920 to 0.999). This shows that the non-linear modeling approach could learn also the significant linear correlation between the 2001 and 2007 IMT-levels (*r* = 0.582; Table S3).

**Table 3:** The single nucleotide polymorphisms (SNPs) predictive of the subjects with 15% lowest and highest IMT levels in 2007.

SNP ID* (rs number)	Gene symbol (HGNC name)	SNP location (Chr region)	Significance† (*p*-value)	Predictive power‡ (%AUC)
rs17672135	FMN2	1q43	0.41	17.5
rs9941339	CDH13	16q24.2-q24.3	0.75	8.75
rs2548861	WWOX	16q23.1	0.14	8.75
rs9939609	FTO	16q12.2	0.69	7.50
**rs693**	APOB	2p24.1	0.53	7.50
rs17222814	ALOX5AP	13q12.3	0.89	7.50
rs1041981	LTA	6p21.33	1.00	7.50
rs9551963	ALOX5AP	13q12.3	0.64	6.25
rs7524102	INTERGENIC	1p36.12	0.77	5.00
rs2516839	USF1	1q23.3	0.13	5.00
rs2301880	WNK1	12p13.33	1.00	5.00
rs7759938	INTERGENIC	6q21	0.12	3.75
rs9479055	C6orf97	6q25.1	0.40	3.75
rs3130340	INTERGENIC	6p21.32	0.11	3.75
rs2553268	WRN	8p12	0.15	2.50

*The SNPs identified also in the previous case-control association studies [9]–[21] are boldfaced.

†The corrected *p*-values larger than one were truncated to unity.

‡The SNPs are arranged according to their contribution to the overall prediction accuracy (AUC).

### Genetic variants predisposing to IMT progression

We next searched explicitly for those factors that are most predictive of the subjects who show extreme progression in their IMT-levels between the two follow-up studies. When applying the machine learning-based procedure to prediction of the subjects with increasing changes in their IMT-levels between the study years 2001 and 2007, the selected SNPs could again systematically increase the predictive power across all the progression risk classes, compared to the accuracy obtained with the conventional risk factors either alone or when combined with the panels of variants identified in the previous case-control studies or in the present study population using single-SNP statistical testing (Figure 2C). In this case, however, the prediction accuracies were not anymore monotonically decreasing functions of the quantile point (*q*). In particular, the 10% risk class was found to be problematic, which could be due to the particular IMT cutoff values used in its quantitative definition. Interestingly, the SNP set most predictive of the IMT progression contained a relatively large number of variants with modest contributions to the predictive power; of these variants, only one was among the established markers identified in the previous case-control studies (Table 4). Even if the IMT progression proved relatively difficult to predict, the many novel markers support the potential and added value of genetic variation, especially when evaluating susceptibility to the most extreme progression risk class (*q* = 5%).

**Table 4:** The single nucleotide polymorphisms (SNPs) predictive of the subjects with 15% lowest and highest IMT changes from 2001 to 2007.

SNP ID* (rs number)	Gene symbol (HGNC name)	SNP location (Chr region)	Significance† (*p*-value)	Predictive power‡ (%AUC)
rs2073658	USF1	1q23.3	0.70	9.40
rs9479055	C6orf97	6qs25.1	0.40	8.55
rs17672135	FMN2	1q43	0.41	8.55
rs9687339	MAST4	5q12.3	0.93	7.69
rs1042713	ADRB2	5q33.1	0.48	7.69
rs2301880	WNK1	12p13.33	1.00	6.84
rs3130340	INTERGENIC	6p21.32	0.11	6.84
rs2476601	PTPN22	1p13.2	0.44	5.13
rs11898505	SPTBN1	2p16.2	0.27	5.13
**rs3798220**	LPA	6q25.3	1.00	5.13
rs10172036	ICOS	2q33.2	0.52	5.13
rs2820037	INTERGENIC	1q43	0.66	4.27
rs2234693	ESR1	6q25.1	0.74	3.42
rs1800896	IL10	1q32.1	0.71	3.42
rs17222814	ALOX5AP	13q12.3	0.89	3.42
rs1801274	FCGR2A	1q23.3	0.75	2.56
rs854560	PON1	7q21.3	0.81	1.71
rs10246939	TAS2R38	7q34	0.80	1.71
rs9594738	INTERGENIC	13q14.11	0.58	1.71
rs1799983	NOS3	7q36.1	0.06	0.855
rs1256049	ESR2	14q23.2	0.46	0.855

*The SNPs identified also in the previous case-control association studies [9]–[21] are boldfaced.

†The corrected *p*-values larger than one were truncated to unity.

‡The SNPs are arranged according to their contribution to the overall prediction accuracy (AUC).

### Epistasis interactions between the predictive variants

**Figure 3:**
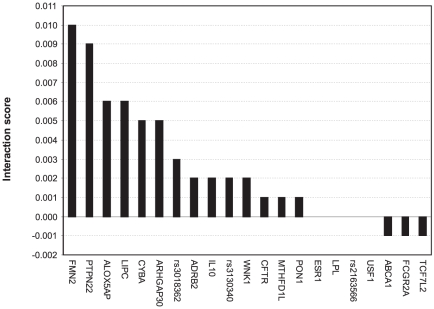
Candidate interaction partners of a variant in USF1 (rs2516839). The candidate SNP-SNP interactions were searched among the variants predictive of the extreme IMT progression (see Table S4). The interaction score for a SNP-pair (*x*,*y*) is 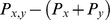, depicting the combined contribution of the SNP-pair to the predictive power (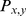), relative to that of the individual SNPs' contributions (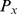 and 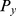). The predictive power was assessed in terms of how much the AUC value changed when the particular SNP or SNP-pair was deleted from the subset of variants. The Gene ID was used as a SNP identifier, where available; otherwise, the rs ID was used instead.

To identify candidate epistasis (or synergistic) interactions between the genetic risk factors, we searched for such pairs of genetic variants that led to the largest drop in the prediction accuracy when removed together from the set of predictive SNPs, relative to the drop resulting from removing either of the variants separately. As a feasibility study, we explored the particular SNP set which was found to be highly predictive of the subjects with the most extreme IMT progression from 2001 to 2007 (Figure 2C, *q* = 5%). When investigating a specific variant (rs2516839) in the upstream stimulatory factor 1 (USF1), a known regulator of the transcription of several cardiovascular-related genes, we identified a number of potential genetic interaction partners of USF1 (Figure 3), including formin 2 (FMN2, rs17672135), protein tyrosine phosphatase, non-receptor type 22 (PTPN22, rs2476601), hepatic triglyceride lipase (LIPC, rs1800588), and arachidonate 5-lipoxygenase-activating protein (ALOX5AP, rs17222814). It is interesting to note that each of these candidate gene-gene interactions originated from different biological processes, indicating that the disease progression and phenotypic heterogeneity is likely due to genetic alterations within multiple molecular pathways (Table S4). Such interactions and pathways may serve as basis for more detailed further studies of the molecular mechanisms and disease networks that predispose to such excess levels of the IMT-progression that can lead to clinical cardiovascular events in the future.

### Evaluation on independent and randomized subject sets

**Figure 4:**
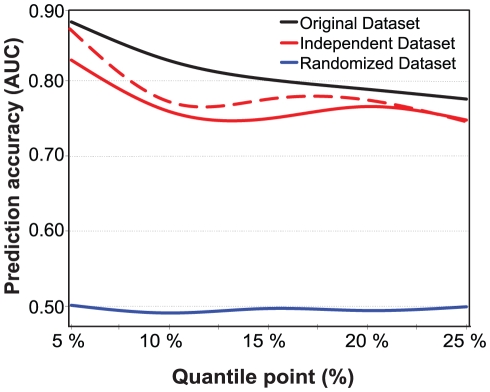
Prediction accuracies on independent and randomized subject sets. The accuracy was defined using the area under the receiver operating characteristic curve (AUC), and the risk classes using the quantile points (5%–25%). The prediction accuracies were evaluated for the baseline IMT risk classes in the independent dataset, in comparison with the cross-validated accuracies obtained in the original dataset using the same IMT thresholds, conventional risk factors and the most predictive SNPs identified with the machine learning-based procedure in the original subject set. The dotted trace shows the effect of deleting those subjects whose IMT level was the same or close to the quantile cut-off value (<0.02 difference in IMT). The randomized datasets were generated by first dividing the original set of subjects into the low- and high-risk classes at random, independent of their IMT-levels, and then repeating the same randomization process 100 times for each of the risk classes. The average AUC level for the various risk classes is reported. None of the 500 randomized datasets produced prediction accuracy higher than that obtained using the most predictive SNPs identified in the original set of subjects.

To further explore the generalization capability of the prediction models estimated and evaluated on the current study subjects, we constructed a separate validation set consisting of those subjects who were filtered out in the initial subject selection because of missing data, but had a complete set of those SNPs identified for the particular risk class (see Figure S1). These new subjects were then split into the classes of ‘low-risk’ and ‘high-risk’ based on the exact same IMT-cutoff values that were used in the original subjects. In general, the results in the independent validation set scaled as expected (Figure 4). Even if the prediction of the new subject classes using those SNPs identified in the original dataset led to decreased prediction accuracies (average decrease in AUC was 0.043), their prediction capability was shown to extend beyond the original subjects, especially for the extreme 5% IMT cases, whereas the 10% risk class again showed poorer performance. A part of the decreased accuracy can be attributed to the sensitivity of the extreme selection strategy to the particular IMT quantile cut-offs being used (the dotted trace). We also repeated the same model building and evaluation framework for randomized datasets, in which subjects were divided into the low- and high-risk classes at random. This resulted in random prediction accuracies (average AUC 0.496), indicating that the high accuracies obtained with the predictive models were not by chance alone (Figure 4). Based on these results, independent and randomized subject sets were found to be useful for controlling the degree of overfitting, even when cross-validation is used in the model building.

## Discussion

The present results demonstrate a predictive relationship between an individual's genotypic variation and early signs of atherosclerosis along with its progression over a 6-year period in our population-based longitudinal follow-up study. The relationship was much stronger with the variants identified using the machine learning-based approach compared to the variants identified using single-locus statistical hypothesis testing procedures either in the present study population or in the previous case-control association studies of clinically manifesting CHD [9]–[21]. This latter finding is in line with a recent observation that the genetic scores, constructed from individual SNPs that met the genome-wide level of statistical significance in earlier GWASs, could not improve the prediction of cardiovascular risk after adjustment for conventional cardiovascular risk factors [41]. Similar observations have been made in the context of other diseases when using such a ‘bottom-up’ approach to building discrimination models [33]. In the present study, rather than exclusively using only those variants with the lowest *p*-values for association, we took here an alternative ‘top-down’ approach to predictive modeling by explicitly searching for all of the genetic and conventional risk factors that positively contribute to the prediction power. It was surprising to note that, among the most predictive variants, there was only a single statistically significant SNP in the present cohort (see Table 2, Table 3, Table 4), supporting the idea that many of the predictive associations are detected much lower down on the ranked list of hits compared to the top hits with the highest statistical support [43]. Ignoring such ‘gray zone’ variants is likely to result in missing an important proportion of the quantitative variation in heritability [44]. The proposed predictive modeling framework therefore complements the statistical class comparison procedures traditionally used during the discovery phase.

We used our longitudinal cohort data of carotid atherosclerosis precursors to implement a class prediction model, with the specific aim to build a multivariate discrimination function, or a classifier [45], which can accurately predict the risk class of a new subject on the basis of a panel of key variants. Sampling of the subjects with increasing carotid IMT levels from our follow-up study provided us with the unique opportunity to investigate the genetic variants contributing to the present and future atherosclerosis risk. Evaluation of the genetic variants predictive of the 2001 IMT risk classes was used here to set a baseline for the prediction accuracies and for the corresponding SNP panels. Medically, it is perhaps most interesting to evaluate the ability to predict the future IMT risk classes as well as the progression of the IMT levels over the time. The determination of the future atherosclerosis risk is analogous to predicting the 2007 IMT risk classes based on the data reflecting the 2001 baseline genetic variants and confounding risk factors. The IMT progression (i.e., difference between the 2007 and 2001 IMT levels) is relevant in that even though an individual may not be considered to be in the risk group in 2007, the rate of change in the IMT levels between the evaluation years is large enough to warrant the subject as still being regarded as being at higher risk. The group with extreme IMT progression therefore represents the set of subjects who would be potential candidates for primary prevention in order to offset their likelihood of developing carotid atherosclerosis in the future. The full set of the SNP-panels predictive of the IMT-levels in the 2001 and 2007 studies, as well as of its relative progression from 2001 to 2007, are listed and characterized in Table S1. The genetic interactions between those variants that were highly predictive of the extreme IMT-progression are further discussed in Text S2.

Those SNPs that were found to be the most predictive of the 15% risk classes of IMT-levels and progression (Table 2, Table 3, Table 4) can be interpreted on the basis of a prior knowledge (Table S5). Most of the SNPs and corresponding genes have earlier been associated with cardiovascular disease risk factors such as low serum HDL-cholesterol and high serum LDL-cholesterol, triglycerides, lipoprotein(a) and apolipoprotein B concentrations (i.e., APOB, LPA, WWOX, ABCA1, USF1, PSRC1, ADRB2), inflammation, inflammatory and immunological factors such as serum CRP and interleukin levels (i.e., CRP, IL18, IL1B, LTA, ALOX5AP, IL10, ICOS, PTPN22), blood pressure, hemodynamics as well as serum asymmetric dimethyl arginine concentrations (DDAH2, WRN, WNK1, CDH13, NOS3), obesity, BMI, metabolic syndrome (FTO, ADRB2), and lipoprotein oxidation (PON1). Most of these SNPs are also linked to different cardiovascular traits, such as coronary artery disease, coronary artery calcification and atherosclerosis plaque areas, myocardial infarction, sudden cardiac death, stroke, as well as having phenotypic relationships with subclinical atherosclerotic traits such as carotid IMT (ESR1, APOB, PON1, USF1, ALOX5AP, ESR2, IL10, FCGR2A). Such associations have been found either alone or by interaction with other genes and clinical or environmental factors, including diabetes mellitus and use of alcohol or smoking [46], [47]. There were also novel IMT-related SNP candidates, earlier associated with bone density (C6orf97 and some intergenic SNPs), revealing possible mechanistic links to bone mineral and calcium metabolism. It is known that morphogenetic proteins and vascular calcification are activated in advanced atherosclerotic plaques [48]–[50]. On the basis of the present results, the same seems to hold true already in the sub-clinical stage of carotid atherosclerosis.

### Limitations of the study and future developments

As with any association study that evaluates the contribution of a large number of candidate variants to a given phenotype, the question of how well the results will generalize to other study populations remains to be studied. This is a potential limitation in all SNP studies regardless of whether the class comparison or class prediction approach is being applied. It is known that associations identified in one population using the single-SNP statistical hypothesis testing procedures may not be detected in other populations in part due to the *p*-values being affected by the confounding factors [29], [51]. Measures which directly evaluate the predictive value of multiple factors, such as AUC-values, can overcome some of these limitations but are not without caveats [32], [33], [52]. Unlike many other class prediction studies that have used the AUC to assess the discrimination accuracy within the given cases and control subjects only, here we used cross-validation both when selecting coherent subsets of the most predictive variants, through feature selection, as well as when evaluating their prediction accuracy, as compared to the subsets of the most significant SNPs. Cross-validation was necessary to avoid a selection bias, which can lead to over-optimistic prediction results and the reporting of a large number of over-fitted genetic variants [45], [53]. The final evaluation of the panels of SNPs was done using an independent subject set to confirm that the reported models also generalize to other sub-populations beyond those used in the initial model estimation and validation. Testing on an independent dataset can also help to resolve any biases that may exist due to the fact that the cross-validation folds are far from independent of one another.

In common with many other SNP-studies, our main objective here was to find out those variants that are the most predictive of the atherosclerosis risk and progression in our follow-up study. When the aim is to obtain high prediction accuracies, the rules for including factors in the discrimination model are different from those when searching for the strongest statistical associations [54]. However, regardless of whether the discoveries come from statistical significance testing or from machine learning-based SNP-selection, the selected variants need to be carefully validated in further studies [55]. These two complementary approaches have also been combined, by building prediction models based exclusively on statistically significant SNPs, but this combined approach has been shown to result in poor classification accuracies [33]. In fact, reasonable increases in the prediction accuracies are often obtained only after including hundreds of top variants, depending on the complexity of the disease phenotype and whether or not cross-validation is utilized [32], [38], [39]. When the aim is class prediction, we believe it is better to make use of those methods that are specifically designed for optimal prediction, together with stringent feature selection and cross-validation, to output modest number of highly predictive and reliable variants for further study [45]. Further evaluation of the prediction power on independent and randomized subject sets was also found to be useful for controlling the degree of over-fitting, as shown in Figure 4, even when systematic cross-validation schemes are being used in the model building process [56], [57].

It was interesting to note here that the simple naïve Bayes classifier performed well in the prediction of the atherosclerosis risk. The conditional independence assumption behind this probabilistic prediction model results in the nominal predictive probabilities that are often unrealistic, in the sense of being very close to either zero or one. Therefore, we followed the standard practice and chose the class with the highest posterior probability. Despite this simplifying assumption, the naïve Bayes classifier generally provided the best prediction results across the various risk classes, compared to other classification models, such as Bayes Nets, Support Vector Machines, or Random Forest (see Text S1 for their comparison). Moreover, because of its simplicity, the naïve Bayes classifier is also computationally more efficient than the other, more complex prediction models, enabling its usage in GWAS meta-analyses as well. These observations are in line with previous works, which have shown that the naïve Bayes classifier can perform well even in the case when there are strong dependencies in the dataset [58]–[60]. In particular, it has proven to be effective in the context of the IMT-phenotype and in SNP-data [61], [62]. Standard filtering procedures, such as those based on the Hardy-Weinberg equilibrium, and other quality control measures implemented during the genotyping can result in severe restrictions on the joint distribution of alleles, enabling them to appear independent of one another, further explaining the good performance of the naïve Bayes classifier. However, other efficient SNP-subset selection methods that go beyond the single-SNP testing, such as those based on penalized maximum-likelihood approach [63], or different filter-wrapper machine learning approaches [31], could be used in the generic modeling framework.

While previous studies have identified sex-related differences in the cardiovascular disease incidence and genetic risk factors [64], the objective of the present study was to demonstrate that a common panel of genetic risk factors can already improve the prediction of subclinical carotid atherosclerosis risk and progression in a general population of young adults. Therefore, we did not stratify the subjects on the basis of any of the conventional risk factors, including sex or age, but the subjects were combined into a single distribution (Figure 1). In the future studies, however, it is possible to divide the heterogeneous population into more homogeneous sub-samples to investigate the relationship between the genetic and conventional risk factors in more controlled settings. Further, pathway and network analyses of such sub-sample-specific genetic variants and their interactions could reveal also underlying similarities or differences in the biological processes and genetic networks [6]. We have previously shown that sub-sampling-based automated procedures can help to detect hidden subject sub-groups that present with similar genetic profiles in genome-wide studies and which may associate with divergent clinical outcomes [65]. An automated subject grouping combined with the predictive modeling framework introduced in the present study could offer possibilities to start developing personalized approaches that make the most of genetic variation together with clinical data to predict individual susceptibility to the initiation and progression of carotid atherosclerosis and other complex diseases. Such experimental-computational approaches may prove to have also clinical utility in the early detection and management of sub-clinical atherosclerosis and other quantitative disorders.

## Materials and Methods

### Subject selection

The Cardiovascular Risk in Young Finns Study is an on-going population-based follow-up study of atherosclerosis precursors from childhood to adulthood [66]. The multi-center study has been carried out in five university hospitals across Finland (Turku, Tampere, Helsinki, Kuopio and Oulu). The baseline cross-sectional study in 1980 included a total of 3,596 children and adolescents, aged between 3–18 years, who were randomly chosen from the national population register [67]. Since then, follow-up studies have been conducted in 1983, 1986, 2001 and 2007, in which the conventional risk factor data have systematically been collected from the individuals participating in those studies. In the two most recent follow-ups in 2001 and 2007, which were used in the present analysis, a total of 2,283 and 2,204 participants were re-examined, comprising the age groups of 24, 27, 30, 33, 36, 39 years and 30, 33, 36, 39, 42, 45 years, respectively; out of these, a total of 1,828 subjects participated both in the 2001 and 2007 follow-up studies [68]. The subjects involved in the cohort provided written consent to be included in the study approved by local ethics committees.

The study cohort for the present analysis was comprised of those subjects who took part in both the ultrasound and the genotyping studies in 2001. The carotid artery intima-media thickness (IMT) was measured from 1,809 subjects in both of the follow-up studies. Genotyping of single nucleotide polymorphisms (SNPs) was based on the DNA collected in 2001. The candidate gene approach was used to explore potentially interesting relationships between several known SNPs and clinical traits. Subjects who had missing values either in their IMT or SNP data in the year 2001 or 2007 were excluded from the present analysis, in order to eliminate their potentially adverse effects on both the reported prediction accuracies and on the selected genetic variants. Due to such stringent subject selection criteria (see Figure S1), the complete data matrices from *n* = 1,027 subjects were used in the search of genetic variants (SNP sets) that are predictive of the atherosclerosis (indexed by IMT) at baseline (2001); of these, *n* = 813 had complete data also in the follow-up study (2007), and could be used when searching for variants predictive of IMT progression (the change from 2001 to 2007).

### Clinical characteristics

In the present analysis, we used the conventional risk factor data from the 2001 follow-up study. The physical examination consisted of the measurement of height, weight, systolic and diastolic blood pressure, and waist circumference [66]. The body mass index (BMI) was calculated by dividing the patients' weight in kilograms by the square of their height in meters. Waist circumference was recorded as the average of two measurements with an accuracy of 0.1 cm. Blood pressure was measured at least three times with a random zero sphygmomanometer, and the average of the three readouts of systolic and diastolic blood pressure was recorded. Lifestyle risk factors, such as smoking, were examined with questionnaires; the subjects who smoked daily were regarded as smokers. For the assessment of serum lipoprotein levels, venous blood samples were drawn after an overnight fast and the serum was separated, aliquoted and stored at −70°C until analysis. Standard enzymatic methods were used for recording the levels of serum total cholesterol, HDL-cholesterol, and LDL-cholesterol, as well as the concentrations of serum triglycerides, apolipoprotein A1 (ApoA1) and B (ApoB) [67], [68].

### Genotyping studies

Genomic DNA was extracted from peripheral blood leukocytes with a commercially available kit (Qiagen Inc., Valencia, CA). The DNA samples collected during the 2001 follow-up study were genotyped as described previously [66], [69]. In the present analysis, we included the panel of 17 SNPs with the highest single-SNP statistical significance in the recent GWASs identifying variants for CHD outcomes and serum lipids [9]–[21], as well as a number of other candidate SNPs listed in the first phase of the international pooling project of cardiovascular cohorts [70]. A total of 108 SNPs with complete genotyping data in the selected subjects were considered here in the predictive modeling; these SNPs are generally related to serum lipid and lipoprotein metabolism, oxidation, cellular lipid metabolism, inflammation, immunological system, cell signaling, cell migration, cell growth, homocystein metabolisms, cellular adhesion and blood coagulation (see Table S1 for the full list of SNPs together with information on their gene annotation and chromosomal location, as well as on associated phenotypes available from previous studies).

### Ultrasound imaging

Ultrasound studies were performed using Sequoia 512 ultrasound mainframes (Acuson Inc., Mountain View, CA, USA), with 13.0 MHz linear array transducers. Exactly the same scanning protocol was used both in 2001 and 2007 studies, as previously described [23]. Briefly, carotid IMT was measured on the posterior (far) wall of the left carotid artery. At least four measurements were taken 10 mm proximal to the bifurcation, and the average of the readouts was recorded. The digitally stored scans were manually analyzed by the same reader both in 2001 and 2007 blinded to the subjects' characteristics. The between-visit coefficient of variation of such IMT measurements was 6.4%, as estimated between two visits that were three months apart [23]. Since the IMT correlates with the risk of atherosclerosis progression and subsequent cardiovascular events [23]–[27], it was used here for stratifying the subjects into gradually increasing risk classes. Being non-invasive in its nature, this measurement can be justified in large populations of healthy subjects, without biases related clinically diagnosed cases and controls [28], making it a convenient quantitative phenotype of atherosclerosis in population-based follow-up studies. The quantitative IMT measurement suffers from a degree of measurement error, which can lead to regression to the mean (Figure S2).

### Predictive modeling

The relative contribution of the conventional and genetic risk factors to the individual IMT levels was investigated by means of a predictive modeling framework, similar to that which we and others have used before [61], [62]. Briefly, the study subjects were first divided into several risk classes according to their IMT levels. Based on the concept of extreme selection strategy [1]–[3], the quantile points, say (1-*q*) and *q*, of the IMT distribution were used to define the low and high risk classes, respectively (see Figure 1). The prediction of whether a subject belongs to the high-risk (*H_q_*) or low-risk (*L_q_*) class was done on the basis of his or her individual SNP data (*S*_1_, …, *S_l_*), whereas clinical characteristics, smoking habits, sex and age were used as confounding risk factors (*C*_1_, …,*C_m_*). A probabilistic prediction model, the so-called naïve Bayes classifier, was used here because of its low computational cost and good performance in previous studies [61], [62], [71]. Mathematically, the predictive classifier can be formulated as a conditional probability of observing the true class *R* (either *H_q_* or *L_q_*) given the genetic and confounding risk factors (the predictors *P*):
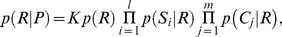
(1)where *K* is a scaling factor independent of the risk class *R*. The *a priori* probabilities 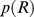 were set to the number of training samples in the low and high classes [71], and for numeric risk factors, the training algorithm estimates the densities 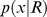 using Gaussian distributions [72] (see Text S1 for more details). The subjects in the test material were then classified by choosing the risk class with the highest *posterior* probability in Eqn (1). The predictive power of different risk factor combinations was assessed with the *k*-fold cross-validation procedure, in which the given sample was divided into *k* distinct subsets of equal sizes, each of which in turn was used as a validation set, to assess how well the results will generalize to new sets of subjects, while the remaining sub-samples were used in the initial training of the prediction model [71]. The final prediction accuracy was reported as the average over the *k* validation rounds (here *k* = 10; see Figure S3).

### Selection of predictive variants

The selection of predictive genetic and conventional risk factors was performed in two-steps, with the aim of identifying a minimal set of informative features for predicting the different risk classes (see Figure S3). The SNP selection was done using a machine-learning-based procedure, similar to the ‘filter-wrapper’ method [73]. The filtering phase starts from the full set of SNPs and uses an entropy-based information gain measure to reduce the high-dimensional search space to the subset of most informative genetic and conventional risk factors (here 40), which could subsequently be traversed thoroughly in the next phase of selection. In the wrapper phase, the best first-based iterative search-and-evaluate algorithm was used to further improve this subset by excluding those factors with least predictive power, using backward search direction, while the backtracking option allows for escaping from local optima [71]. The predictive power of the selected factor combinations was assessed using the naïve Bayes classifier, run with a 5-fold cross-validation to avoid potential selection bias, and the final prediction accuracy was evaluated using external 10-fold cross-validation (see Figure S3). The predictive modeling and risk factor selection was carried out with the Weka data mining platform (version 3.7; University of Waikato, New Zealand) [71].

### Assessment of prediction accuracy

The predictive accuracy of the classifiers, constructed using either the *p*-value-based selection of the most significant SNPs or the machine-learning-based selection of the most predictive SNP-sets, was assessed using the receiver operating characteristic (ROC) analyses; ROC curves characterize the relative trade-off between true positive rate (sensitivity) and false positive rate (1 – specificity) of a classifier over the whole range of discrimination thresholds [32], [33], [71]. The overall accuracy of a classifier was summarized using the area under the ROC curve (AUC) measure; for an ideal classifier, AUC = 1, whereas a random classifier obtains an AUC = 0.5 on average [52], [61], [71]. The relative predictive power of each individual SNP or SNP-SNP interaction was assessed in terms of the change in AUC level when the particular SNP (say *x*) or the SNP-pair (*x*,*y*) was deleted from the selected set of variants (denoted by 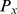 and 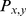, respectively). The interaction score for detecting epistasis effects was defined as 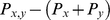, resembling additive definition of genetic interactions based on single and double-deletion experiments in model organisms [74]. The AUC-values were calculated using the Weka platform (version 3.7; University of Waikato, New Zealand) [71].

### Statistical procedures

The level of statistical association of single SNPs with the IMT-classes was assessed by determining the genotypic probabilities (*p*-values), on the basis of the 2×3 contingency matrix that contains the counts of the three genotypes among the low-risk and high-risk subjects [75]. Computationally efficient calculation of the exact *p*-values for each individual SNP was carried out with the ExactFDR software [76]. The Pearson correlation coefficient was used to assess the linear association between the various conventional risk factors and IMT-levels or changes. These *p*-values were adjusted for multiple testing using the Bonferroni correction. Although it is known that this correction may be conservative, especially when the test statistics are dependent, it provides an effective means for ensuring that the findings deemed most significant are not by chance alone when many hypotheses are being tested simultaneously. Differences in the distributions of the IMT-levels or changes between sub-populations were assessed using the Kolmogorov-Smirnov *D*-statistic, which is based on the maximal vertical distance between the two distributions. The statistical analyses were carried out with the SPSS Statistics software (version 17.0; SPSS Inc., Chicago, IL, USA) and with the statistical computing platform R (http://www.rproject.org/).

## Supporting Information

Figure S1 The selection of the subjects and SNPs for the original dataset and for the independent validation set from the Cardiovascular Risk in Young Finns Study cohort. The white entries represent missing data points and their corresponding SNPs and subjects were removed by the final dataset which is represented by the completely shaded box on the upper left hand corner. The first inclusion criterion for the subjects was that they must have complete data for the set of 17 variants that have previously been associated with cardiovascular events (Established SNPs, the yellow submatrix). After that, the set of SNPs was extended gradually, to incorporate as many subjects as possible with complete SNP data. This selection procedure resulted in a sub-matrix of 1027 subjects and 108 SNPs that were used here when searching for the variants predictive of the severity and progression of sub-clinical atherosclerosis (Candidate SNPs, the blue submatrix). In order to create the independent validation dataset, the set of patients who were not part of the original 1027 subject subset were searched for those individuals who had complete data for all of the SNPs involved in a particular predictive model (Predictive SNPs). The number of patients, *n*, in each of the independent sets varied according to the particular risk class the validation set was created in relation to (*n* = 103, 222, 300, 351 and 423, for the 5%–25% risk classes, respectively).
(0.17 MB PDF)

Figure S2 Scatter plots of the IMT levels (A) in 2001 and 2007, and (B) with 2001 and the change in value between 2001 and 2007, both fitted with their respective linear correlation models (black lines). The plots are marked with two sets of vertical lines indicating the numerical IMT cutoff values used to select the 5% (red solid lines) and 15% (blue dashed lines) extreme quantiles and to split the subjects into the low-risk and high-risk classes. Although regression to the mean is observed, as was expected, it can be seen that the 15% extreme value class contains both increasing and decreasing IMT values, making it a unique situation in which the classifier must try to predict different IMT change directions within individual risk classes.
(0.53 MB PDF)

Figure S3 Schematic illustration summarizing the model building and evaluation procedure. Implementation and evaluation of the machine learning-based feature selection algorithm, compared to using the single-SNP *p*-values (the right-hand track). The aim of the algorithm was to select the subset of genetic factors (SNPs) and conventional risk factors (CRFs) from the filtered dataset that were the best predictors of the risk classes, determined separately for 2001 and 2007 IMT levels (two follow-up points), as well as for its progression between 2001 and 2007 (IMT progression). The low-risk and high-risk were defined based on the gradually increasing quantiles of the pooled IMT distribution (*q* ranges from 5% to 25%). The most significant SNPs, determined using single-SNP statistical testing for the same risk classes, were used as a reference SNP selection approach in the evaluations.
(0.12 MB PDF)

Table S1 The SNPs explored in the present study, together with information on their gene annotation and chromosomal location (from the dbSNP database), and on associated phenotypes as available from the existing studies (listed in references). Established SNPs refer to those 17 variants identified in the previous CHD case-control association studies. The other columns indicate whether the SNPs were considered predictive of the various IMT risk classes.
(0.10 MB XLS)

Table S2 The statistical significance (p-value) calculated for each of the individual SNPs, depicting their degree of association with the various IMT risk classes in 2001, 2007, and with the IMT changes from 2001 to 2007.
(0.08 MB XLS)

Table S3 Pairwise correlations between the conventional risk factors and with the IMT levels in 2001, 2007, and progression from 2001 to 2007.
(0.05 MB XLS)

Table S4 Molecular pathways and biological processes of the genetic variants predictive of the most extreme 5% IMT change from 2001 to 2007.
(0.04 MB XLS)

Table S5 The interpretation of the SNPs most predictive of the 15% IMT risk classes in 2001, 2007, and of its progression from 2001 to 2007.
(0.04 MB XLS)

Text S1 Details of how Weka platform was used in the prediction studies.
(0.24 MB PDF)

Text S2 Supporting discussion text.
(0.05 MB PDF)
