# Supplementary material for: Genetic Variants and Their Interactions in the Prediction of Increased Pre-Clinical Carotid Atherosclerosis: The Cardiovascular Risk in Young Finns Study
Source: PLoS Genet. 2010 Sep 30;6(9):e1001146. doi: 10.1371/journal.pgen.1001146 (PMC2947986; doi:10.1371/journal.pgen.1001146)
Supplement: Figure S1 — The selection of the subjects and SNPs for the original dataset and for the independent validation set from the Cardiovascular Risk in Young Finns Study cohort. The white entries represent missing data points and their corresponding SNPs and subjects were removed by the final dataset which is represented by the completely shaded box on the upper left hand corner. The first inclusion criterion for the subjects was that they must have complete data for the set of 17 variants that have previously been associated with cardiovascular events (Established SNPs, the yellow submatrix). After that, the set of SNPs was extended gradually, to incorporate as many subjects as possible with complete SNP data. This selection procedure resulted in a sub-matrix of 1027 subjects and 108 SNPs that were used here when searching for the variants predictive of the severity and progression of sub-clinical atherosclerosis (Candidate SNPs, the blue submatrix). In order to create the independent validation dataset, the set of patients who were not part of the original 1027 subject subset were searched for those individuals who had complete data for all of the SNPs involved in a particular predictive model (Predictive SNPs). The number of patients, n, in each of the independent sets varied according to the particular risk class the validation set was created in relation to (n = 103, 222, 300, 351 and 423, for the 5%–25% risk classes, respectively). (0.17 MB PDF) [file pgen.1001146.s001.pdf]

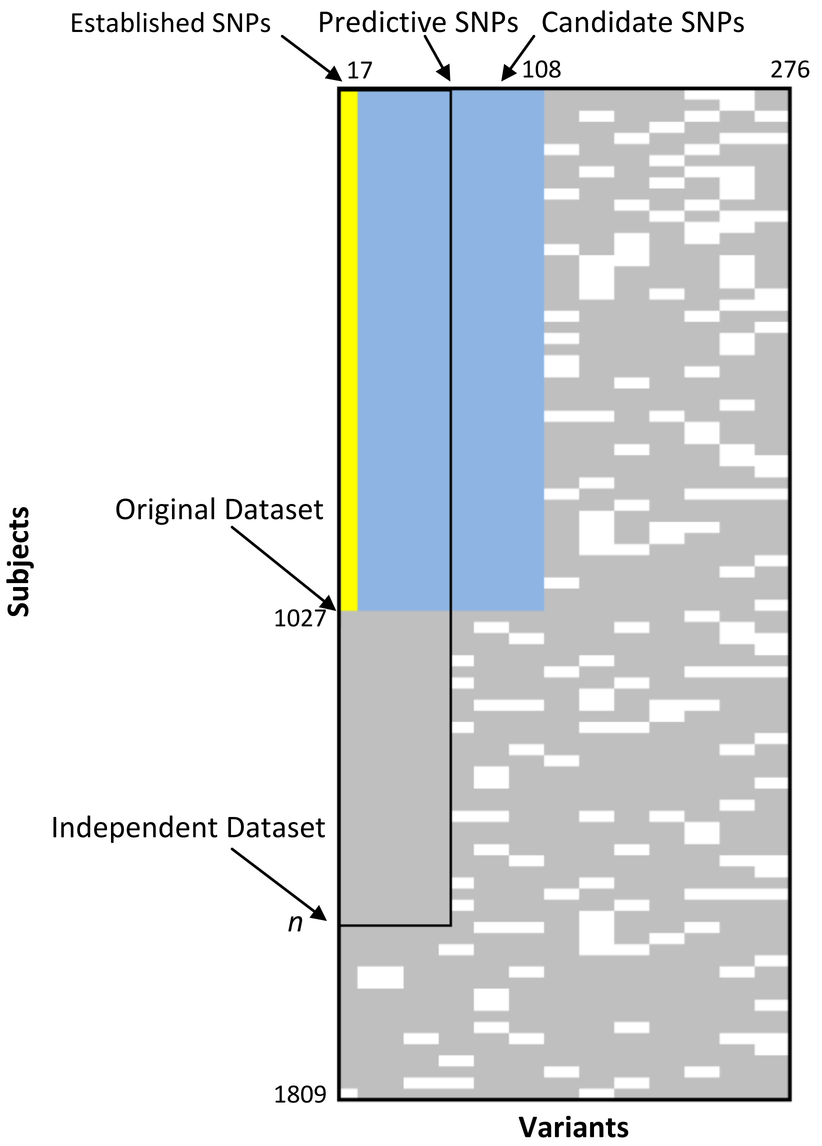

Supplementary Figure 1. The selection of the subjects and SNPs for the original dataset and for the independent validation set from the initial 1809×276 data matrix. The white entries represent missing data points and their corresponding SNPs and subjects were removed by the final dataset which is represented by the completely shaded box on the upper left hand corner. The first inclusion criterion for the subjects was that they must have complete data for the set of 17 variants that have previously been associated with cardiovascular events (Established SNPs, the yellow submatrix). After that, the set of SNPs was extended gradually, to incorporate as many subjects as possible with complete SNP data. This selection procedure resulted in a sub-matrix of 1027 subjects and 108 SNPs that were used here when searching for the variants predictive of the severity and progression of sub-clinical atherosclerosis (Candidate SNPs, the blue submatrix). In order to create the independent validation dataset, the set of patients who were not part of the original 1027 subject subset were searched for those individuals who had complete data for all of the SNPs involved in a particular predictive model (Predictive SNPs). The number of patients, *n*, in each of the independent sets varied according to the particular risk class the validation set was created in relation to (*n*=103, 222, 300, 351 and 423, for the 5%-25% risk classes, respectively).
