# Supplementary material for: Genetic Variants and Their Interactions in the Prediction of Increased Pre-Clinical Carotid Atherosclerosis: The Cardiovascular Risk in Young Finns Study
Source: PLoS Genet. 2010 Sep 30;6(9):e1001146. doi: 10.1371/journal.pgen.1001146 (PMC2947986; doi:10.1371/journal.pgen.1001146)
Supplement: Figure S2 — Scatter plots of the IMT levels (A) in 2001 and 2007, and (B) with 2001 and the change in value between 2001 and 2007, both fitted with their respective linear correlation models (black lines). The plots are marked with two sets of vertical lines indicating the numerical IMT cutoff values used to select the 5% (red solid lines) and 15% (blue dashed lines) extreme quantiles and to split the subjects into the low-risk and high-risk classes. Although regression to the mean is observed, as was expected, it can be seen that the 15% extreme value class contains both increasing and decreasing IMT values, making it a unique situation in which the classifier must try to predict different IMT change directions within individual risk classes. (0.53 MB PDF) [file pgen.1001146.s002.pdf]

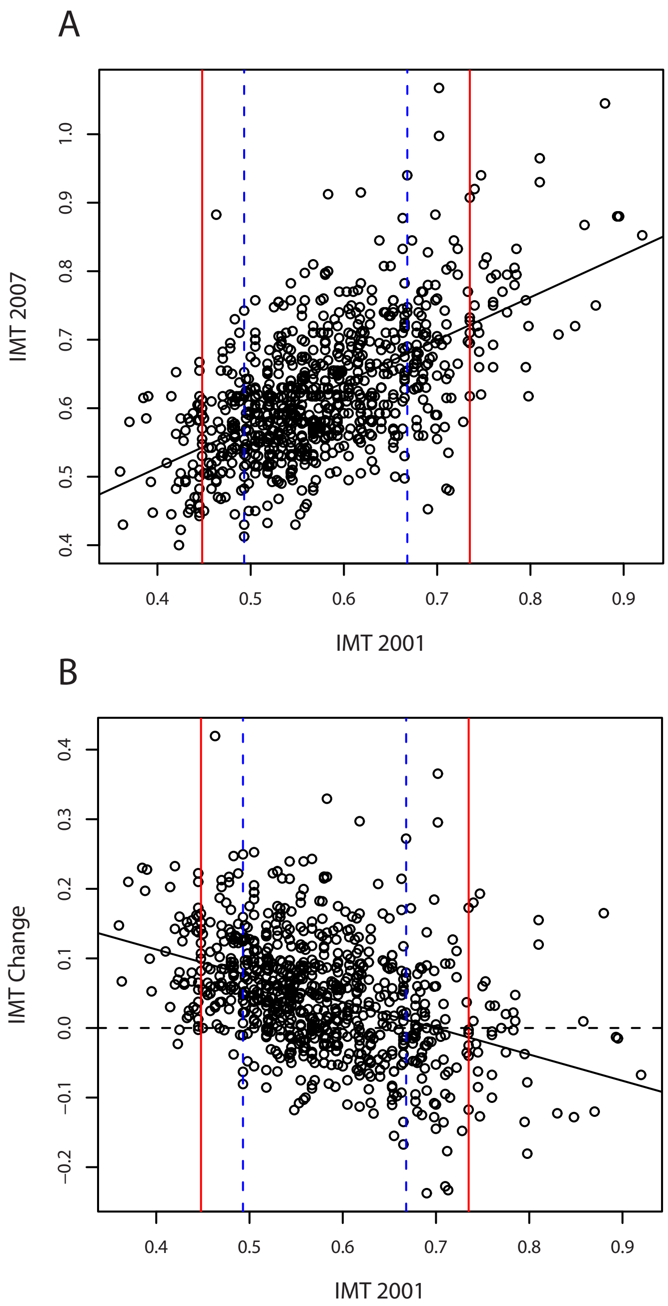

Supplementary Figure 2. Scatter plots of the IMT levels (A) in 2001 and 2007, and (B) with 2001 and the change in value between 2001 and 2007, both fitted with their respective linear correlation models (black lines). The plots are marked with two sets of vertical lines indicating the numerical IMT cutoff values used to select the 5% (red solid lines) and 15% (blue dashed lines) extreme quantiles and to split the subjects into the low-risk and high-risk classes. Although regression to the mean is observed, as was expected, it can be seen that the 15% extreme value class contains both increasing and decreasing IMT values, making it a unique situation in which the classifier must try to predict different IMT change directions within individual risk classes.
