# Supplementary material for: Genetic Variants and Their Interactions in the Prediction of Increased Pre-Clinical Carotid Atherosclerosis: The Cardiovascular Risk in Young Finns Study
Source: PLoS Genet. 2010 Sep 30;6(9):e1001146. doi: 10.1371/journal.pgen.1001146 (PMC2947986; doi:10.1371/journal.pgen.1001146)
Supplement: Figure S3 — Schematic illustration summarizing the model building and evaluation procedure. Implementation and evaluation of the machine learning-based feature selection algorithm, compared to using the single-SNP p-values (the right-hand track). The aim of the algorithm was to select the subset of genetic factors (SNPs) and conventional risk factors (CRFs) from the filtered dataset that were the best predictors of the risk classes, determined separately for 2001 and 2007 IMT levels (two follow-up points), as well as for its progression between 2001 and 2007 (IMT progression). The low-risk and high-risk were defined based on the gradually increasing quantiles of the pooled IMT distribution (q ranges from 5% to 25%). The most significant SNPs, determined using single-SNP statistical testing for the same risk classes, were used as a reference SNP selection approach in the evaluations. (0.12 MB PDF) [file pgen.1001146.s003.pdf]

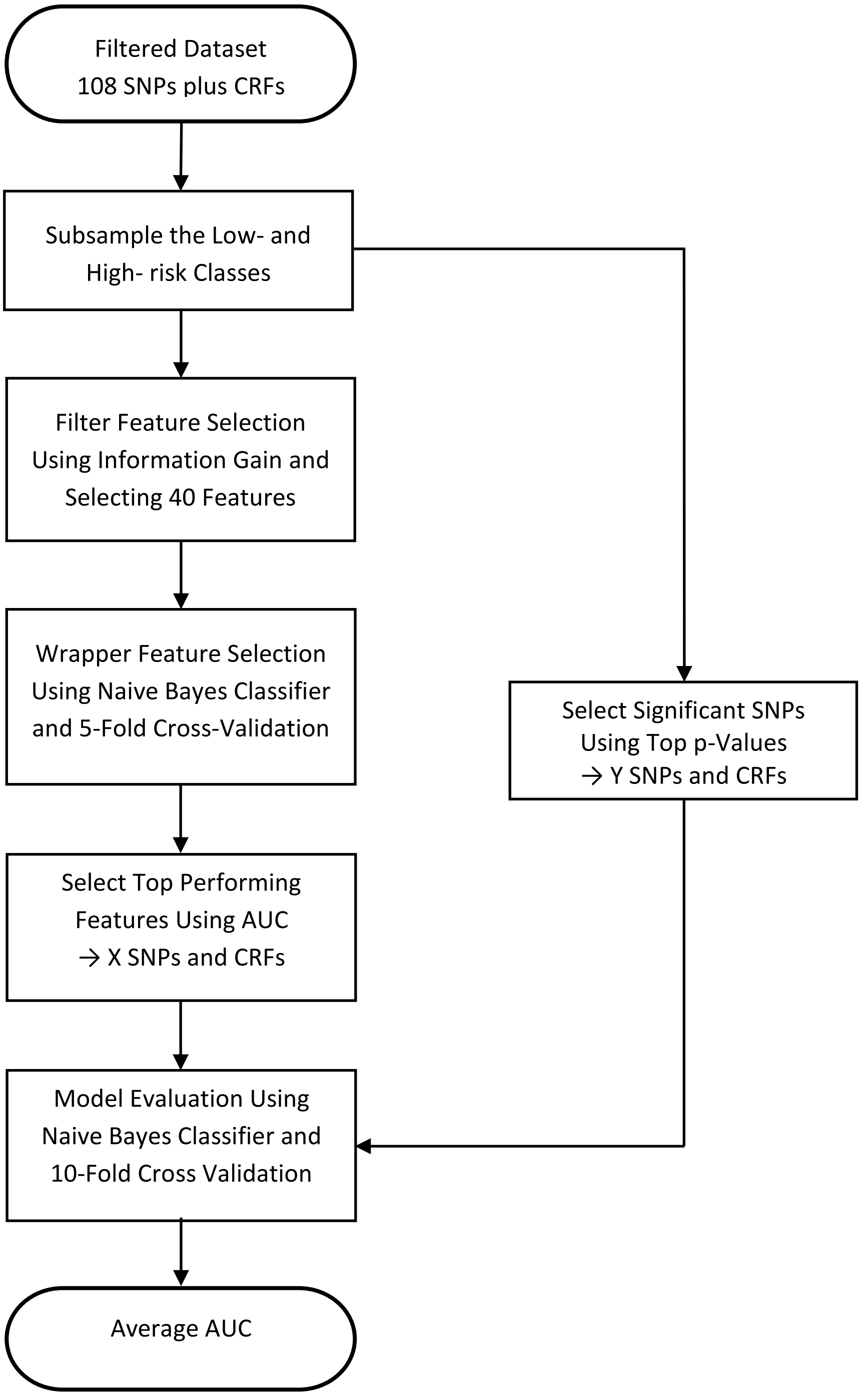

Supplementary Figure 3. Implementation and evaluation of the machine learning-based feature selection algorithm, compared to using the single-SNP *p*-values (the right-hand track). The aim of the algorithm was to select the subset of genetic factors (SNPs) and conventional risk factors (CRFs) from the filtered dataset that were the best predictors of the risk classes, determined separately for 2001 and 2007 IMT levels (two follow-up points), as well as for its progression between 2001 and 2007 (IMT progression). The low-risk and high-risk were defined based on the gradually increasing quantiles of the pooled IMT distribution (*q* ranges from 5% to 25%). The most significant SNPs, determined using single-SNP statistical testing for the same risk classes, were used as a reference SNP selection approach in the evaluations.
