# Supplementary material for: Genetic Variants and Their Interactions in the Prediction of Increased Pre-Clinical Carotid Atherosclerosis: The Cardiovascular Risk in Young Finns Study
Source: PLoS Genet. 2010 Sep 30;6(9):e1001146. doi: 10.1371/journal.pgen.1001146 (PMC2947986; doi:10.1371/journal.pgen.1001146)
Supplement: Text S1 — Details of how Weka platform was used in the prediction studies. (0.24 MB PDF) [file pgen.1001146.s009.pdf]

Supporting text Sebastian Okser *et al.*

Genetic Variants and Their Interactions in the Prediction of Increased Pre-clinical Carotid Atherosclerosis: The Cardiovascular Risk in Young Finns Study

Predictive modeling with the Weka data mining platform

Weka software (version 3.7.0) [1] was primarily used for implementing machine learning functionality onto the datasets that had been prior-filtered for completeness. A number of different filters and classifiers were initially tested and utilized to implement the feature selection and to gauge the performance of the scoring metrics on the dataset. The purpose of the filters was to create a manageable sized dataset that would be free of the biases that could be present from the first analysis of the data. Examples of these biases include that when deciding the cutoff values for the subjects who would be considered either 'Risk' or 'No Risk', the data was sorted based on the numerical IMT values. This sorting could create biases that possibly result in non-random selection of the patients in the different folds of the cross-validation. The flow chart of the steps of the overall data mining process is provided in Figure S3. The following sections describe these steps in more detail.

Selecting informative genetic and conventional risk factors

The first step was to filter out uninformative features (referred to as 'attributes' in Weka) that had originally been kept for reference reasons. Examples of such uninformative attributes are identifier codes for different years when the clinical and diagnostics characteristics were conducted. When splitting the dataset into the folds that are required for cross-validation, the data is stratified and it conserves the proportion of cases to controls. In order to assure that a representative cross-section of the study cohort was included in each fold, the data was first randomized. The next step was to perform an information gain attribute selection on the dataset. This information gain filter is a form of supervised learning and is an option in 'attribute selection' filter in Weka, in which those features that have maximal entropy are ranked higher than those features with lower entropy. During this step, the top forty features in terms of the entropy metric were chosen and the remaining features were deleted.

A further attribute selection was then performed to implement a wrapper-based feature selection. This used the wrapper 'attribute selection' filter in Weka, performed around the particular classifier that was being tested, i.e. Naïve Bayes, utilizing a backwards selection method. This attribute selection used a 5-fold cross validation to select the optimal subset of variables. During this cross-validation, the dataset was stratified to split it into five sets of equal size, maintaining the original proportion of cases to controls. The learning machine was trained on four of the five folds and then tested on the remaining fold. This was done until each fold had been treated as the test fold exactly once. The results were then averaged over the five folds to determine the final results. When applying the wrapper, a backwards selection search method used a Best First search algorithm with a search termination of five. The search termination allows the backtracking of up to five steps to search for better scoring attribute subsets. The Best First search algorithm searches the samples space for attribute sets by the means of a greedy hill-climb algorithm which starts with a random solution and gradually makes minor changes to this solution until an optimal one has been located.

Evaluating the performance of predictive classifier models

After the optimal subset of attributes had been selected, the next step was to implement the classifier and to test its ability to predict the classes based on the attribute subset that was selected using the two-step feature selection. This was done using both cross-validation in the original dataset and a training/test set implementation for the independent validation dataset. The first experiment was conducted by the use of a 10-fold cross-validation which automatically split the dataset into ten folds. These folds were stratified in order to conserve the proportion of cases to controls that were present in the original dataset. The classifier was trained on nine of the folds and then tested on the remaining fold. This was done until each fold had been treated as the test fold. The values of the scoring metrics for each of the 10 folds were then averaged together to compute the final accuracy values for the classifier. In the case of the training/test set implementation, the independent dataset was created as described in the Supporting Figure S1. The classifier was trained on the original training set (the same set that had been prior split up into the 10 folds), and then used to classify the subjects in the newly created independent subject set as the validation set.

Our aim here was to evaluate the prediction capability of the panels of SNPs identified using the feature selection strategy. Our model building and evaluation process incorporated cross-validation both in the selection of a modest subset of consistently predictive genetic variants, through feature selection, as well as in the evaluation of their prediction accuracy, as compared to the significant SNPs (Figure S3). Cross-validation was necessary here to avoid selection bias and reporting of large number of variants that are over-fitted to the training data only. Having too many features in the prediction model may lead to negative result as it increases the probability of genetic masking between the individual SNPs. The cross-validation run for the classifier performance helped to demonstrate, that for each of the folds, the same set of SNPs was performing as expected on different folds. The final evaluation of the panels of SNPs was done using an independent validation set, which can best assess the generalization capability of both the model structure and of the variants selected. Testing on an independent dataset also helps to resolve any biases there may exist in the original k-fold cross-validation because of the fact that the folds are far from independent of one another.

A 5-fold rather than a 10-fold cross-validation was used for the feature selection as a result of the algorithm being designed to be scalable also for complete genome scans. These scans will contain over 500,000 attributes, producing 2^n different feature subsets, where *n* is the number of features in the dataset, which can potentially be evaluated by the search algorithm. This large size yields computational problems and in an attempt to alleviate the number of runs it was decided that it would be best to limit the number of folds that were used during the feature selection stage. The evaluation of the performance of the classifier on the selected feature subset incorporated a 10-fold cross-validation, as the 10-fold is only run on a single feature subset creating a practical solution that maximizes the independence of the different folds. A similar procedure has been used in previous works with high computational complexity [2].

Initial comparison among representative prediction models

To decide which classifier to use, various classifiers were examined for their prediction potential when adapted to the two-step feature selection. This two-step algorithm first applied an information gain filter to rank and select the top 40 features, followed by a wrapper-based feature selection where the attributes are selected based on the features that maximized their

AUC score for the particular classifier being used. The classifiers that were tested were based on the results of similar studies. This resulted in the examination of four different classifiers, representing the usual range of classifiers that are implemented for similar experiments. These were Naïve Bayes, Bayes Nets, Support Vector Machines (SMO) and Random Forest.

Each of these classifiers was tested on their ability to work efficiently when implemented with the two-step feature selection method. In deciding which classifier would be optimal for the SNP-based prediction of the IMT risk classes, each of these classifiers was applied to different risk classes in order to gauge its performance both in terms of AUC and its scalability. While certain classifiers may perform well on smaller sets, if they fail to scale to larger sets it is likely that the accuracy can be due to severe overfitting. We used the 5% and the 15% risk classes to test the learning machines ability to meet to two prior conditions.

**5% Risk Class**		**15% Risk Class**	
**Classifier Used**	**ROC Area**	**Classifier Used**	**ROC Area**
Naive Bayes	0.879	Naive Bayes	0.802
Bayes Net	0.880	Bayes Net	0.786
SMO	0.739	SMO	0.740
Random Forest	0.733	Random Forest	0.750

It was considered important that the chosen classifier has an AUC performance of greater than or equal to 0.8 in order to represent an accurate predictor [3]. Based on this threshold, two choices remained based on the above results in the 5% risk class: Naïve Bayes and Bayes Net. While the AUC score of the Bayes Net narrowly outperformed the Naïve Bayes classifier in the 5% set, its rapid decrease in the 15% set led us to deduce that a more stable classifier would be needed for the various risk classes. The Naïve Bayes classifier scaled as expected throughout the 5-25% classes, while still allowing a quick and efficient means of making the class predictions. While we chose the Naïve Bayes classifier as the final means of class prediction, many other alternative statistical or predictive models could be used instead.

Predicting IMT classes using genetic and conventional risk factors

The conventional Naïve Bayes classifier traditionally constructs the prediction model based solely on categorical data through the application of Bayes Rule to compute the probability of a given class, given the observed values for the attributes that are being used for the prediction. Therefore, the conventional Naïve Bayes implementation can be used when the categorical SNP attributes were used to predict the IMT-based risk classes. In Weka, the Naïve Bayes classifier is implemented as follows [4,5]:

Given an attribute X with attribute value vectors $(x_1, \ldots x_n)$ and an outcome class of C that has individual class labels represented by c, the Naïve Bayes classifier assumes that $x_1, \ldots, x_n$ are conditionally independent of one another. The Bayes' theorem implies that:

$$P(C = c|X = x) = \frac{P(X = x|C = c)P(C = c)}{P(X = x)},$$

where $P(C = c)$ can be estimated based on the training data supplied. Since it is assumed that the attributes are conditionally independent, it can be shown that

$$P(C = c|X = x) \sim P(C) \prod_{i} P(X_i = x_i|C = c).$$

The classifier used for the SNP data did not implement any secondary options such as kernel estimators or supervised discretization of numerical variables. In addition to the categorical SNP attributes, we also used numerical conventional risk factors, such as BMI or blood pressure. This created the challenge of combining both discrete and numeric attributes to build the classifier. This is not an intrinsic task of the Naïve Bayes predictor but its capability is automatically built into the Weka implementation which constructs the classifier by first assuming that all numeric attributes follow a Gaussian probability distribution and it then calculates the mean and standard deviation for each feature, which are used for the estimation of the class.

Briefly, Weka calculates the Gaussian probability density function using the known mean and standard deviation, which in turn is used to determine the risk class. This is done for all of the classes and the numeric attributes that are present in the dataset. If missing values are present for any of the confounding variables, Weka then bases the mean and the standard deviation only on the values that are present. It approximates $P(C|X)$ where C is the class variable and X are the numerical attribute variables through the following [5,6].

For each of the class values of c it can be assumed that the conditional probability of X given C is represented by:

$$P(X = x|C = c) = g(x: \mu_c, \sigma_c),$$

where g is the Gaussian probability density function:

$$g(x: \mu_c, \sigma_c) = \frac{1}{\sqrt{2\pi}\sigma} e^{\frac{(x-\mu)^2}{2\sigma^2}},$$

with mean μ and variance σ. The mean $\mu_c$and variance σ_c are estimated from the values of X in the instances for which $C = c$ simply through unbiased estimates

$$\mu_c = \frac{1}{\#c} \sum_{i=1, c_i=c}^{n} x_i,$$

$$\sigma_c^2 = \frac{1}{\#c - 1} \sum_{i=1, c_i=c}^{n} (x_i^2 - \mu_c^2),$$

where $\#c$ is the number of instances in the dataset where $C = c$.

For further literature on how to calculate pseudo-Bayes estimates in discrete datasets, interested readers are referred to [7].

References

1. Hall M, Frank E, Holmes G, Pfahringer B, Reutemann P, Witten IH (2009) The WEKA Data Mining Software: An Update; SIGKDD Explorations, Volume 11, Issue 1: 10-18.
2. Saeys Y, Degroeve S, Aeyels D, Rouzé P, Van de Peer, Y (2004) Feature selection for splice site prediction: A new method using EDA-based feature ranking. BMC Bioinformatics 5:64-75.
3. Kraft P, Wacholder S, Cornelis MC, Hu FB, Hayes RB, et al. (2009) Beyond odds ratios: communicating disease risk based on genetic profiles. Perspective. Nat Rev Genet 10: 264-269.
4. Long N, Gianola D, Rosa GJ, Weigel KA, Avendaño S (2009) Comparison of classification methods for detecting associations between SNPs and chick mortality. Genet Sel Evol 41: 18.
5. Witten IH, Frank E (2005) Data Mining: Practical Machine Learning Tools and Techniques, 2nd edition. San Francisco: Morgan Kaufmann Publishers.
6. John G, Langley P (1995) Estimating continuous distributions in Bayesian classifiers. Proceedings of the Eleventh Conference of Uncertainty in Artificial Intelligence: 338-345.
7. Bishop YM, Fienberg SE, Holland PW (1975) Discrete Multivariate Analysis: Theory and Applications, Cambridge, Mass., The MIT Press: Chapter 12, pp. 401-434.
