# Supplementary material for: Genetic Variants and Their Interactions in the Prediction of Increased Pre-Clinical Carotid Atherosclerosis: The Cardiovascular Risk in Young Finns Study
Source: PLoS Genet. 2010 Sep 30;6(9):e1001146. doi: 10.1371/journal.pgen.1001146 (PMC2947986; doi:10.1371/journal.pgen.1001146)
Supplement: Text S2 — Supporting discussion text. (0.05 MB PDF) [file pgen.1001146.s010.pdf]

Genetic Variants and Their Interactions in the Prediction of Increased Pre-clinical Carotid Atherosclerosis: The Cardiovascular Risk in Young Finns Study

Prediction of atherosclerosis risk and progression

The discovery of millions of DNA markers has made it possible to identify genetic loci for complex quantitative traits that are influenced by multiple genes of small effect size, instead of focusing solely on qualitative monogenic disorders using case-control designs [1]. Results that are emerging from the GWASs indicate that multiple genes are involved in cardiovascular disorders, implicating that their genetic liability is distributed quantitatively rather than qualitatively. Although the traditional statistical significance testing procedures have provided important biological insights, it has become clear that many of the true associations are detected much lower down on the ranked list of hits, compared to the top hits with the most statistical support [2]. Ignoring the SNPs in the 'gray zone' is likely to result in missing an important proportion of the quantitative variation in heritability [3]. This may partly explain the fact that even though the GWASs have turned up dozens of variants associated with cardiovascular risk, these have had a disappointingly small contribution to the prediction of even clinical CHD outcomes [4-9], not to speak of explaining the pre-clinical stages of cardiovascular disease, such as arthrosclerosis risk and progression [10,11]. Therefore, in contrast to using the statistical class comparison approaches, which aim to determine whether the genetic profiles are different between the given classes of subjects, we took here a machine learning-based class prediction approach, with the specific aim to build a multivariate discrimination function (or a classifier) [12], which can accurately predict the risk class of a new subject on the basis of a panel of key variants.

Epistasis interactions between predictive genetic factors

Most genes work together and it is therefore likely that the effects of one gene on the heritability cannot be revealed without knowing the effects of the other genes [3]. This is an example of epistasis, in which either one gene masks the effect of another or several genes

work together [13,14]. However, due to unknown interactions with other genetic and/or environmental factors, most of the gene-gene interactions are beyond the reach of the standard single-SNP statistical tests [15,16]. In the context of GWASs, efforts to find such epistatic effects generally require good up-front guesses about the interacting partners [3]. The machine learning-based predictive modeling approach provides a natural framework to simultaneously handle the hidden interactions among the genetic and other risk factors, since it searches for composite sets of both genetic and conventional risk factors that positively contribute to the predictive power. Exploring the effects of deleting either individual factors or factor pairs from the predictive sets provides a simple yet effective post-processing option to detect candidate gene-gene or gene-environment interactions. Our definition of the interaction score closely resembles the classic definition of epistasis involving single and double-deletion experiments in model organisms [17]. The calculation time of the interaction scores among the subsets of most predictive variants is independent of the total number of SNPs genotyped, making it possible to exhaustively search and prioritize the most promising candidate pairs of variants that could be subsequently studied in more detail using, for instance, established statistical models [16,18]. Eventually, functional studies are needed to confirm in more detail whether a statistical or predictive interaction also encodes a true biological interaction or an epistatic effect between the selected interaction partners [16-18].

As an example case, we studied here the candidate interaction partners of a particular variant in the USF1 gene, which is known to regulate the transcription of a number of different cardiovascular-related genes and is well established as a gene associated with familial combined hyperlipidemia, a condition increasing the risk for coronary heart disease [19]. In particular, the specific variant under study (rs2516839) has recently been associated with the presence of several types of atherosclerotic lesions and risk for sudden cardiac death [19]. To provide further insights into its potential interaction partners, we explored SNP-SNP interactions in the panel of variants highly predictive of the 5% class of extreme IMT-progression (Figure 3). In addition to the three variants in the genes FMN2 (rs17672135), LIPC (rs1800588), and ALOX5AP (rs17222814), which have already been linked to many cardiovascular disease-related phenotypes, such as IMT, CHD and HDL-cholestrol [20-22], also a variant in the gene PTPN22 (rs2476601) showed an interaction score higher than that expected by the additive effects of individual deletions of the two variants separately (as

captured by the interaction score). These two genes, USF1 and PTPN22, are located on distinct chromosomal neighborhoods and they participate in biological processes that are distinct from each other (Table S4), supporting the diversity of the quantitative disorder also at the level of genomic location and biological pathways. In recent case-control studies, the same PTPN22 variant has been associated with many complex diseases, such as type 1 diabetes, Crohn's disease, and rheumatoid arthritis [23-25]. Its potential role in the progression of pre-clinical atherosclerosis into cardiovascular disease conditions warrants further follow-up studies in those subjects that will present with diagnostic symptoms later in their lives.

Limitations of the study and future developments

To reduce the risk of model over-fitting, which can lead to over-optimistic prediction results, we used here a stringent two-step feature selection procedure that effectively limits the number of either genetic or conventional risk factors that are used in the final prediction models, in accordance with our objective of finding a minimal subset of non-redundant factors that are the most predictive of the risk classes. This selection procedure also highlighted only a subset of the conventional risk factors due to their strong correlation structure (Table S3); in particular, while the age of the subjects was found to be a highly predictive factor in predicting IMT-levels in 2001 and 2007, it was not considered so important when predicting the IMT progression. Therefore, although the actual risk estimates and AUC-values observed here are unlikely to extrapolate to other study populations, we believe that this limitation did not affect our key finding that already a modest panel of selected SNPs can improve the prediction accuracy of the IMT-based risk and progression classes beyond that obtained with the conventional risk factors. The predictive accuracy also remained high in an independent validation set of subjects within the same population cohort (Figure 4). Whether or not similar improvements and genetic variants are also observed in populations with different subject characteristics remain to be studied using, for instance, meta-analyses, where the prediction models are trained using independent subsets from multiple population cohorts with different genetic backgrounds.

A more technical limitation of the present evaluation procedure concerns the subject classification. In absence of established diagnostic thresholds for the IMT-levels, we simply divided the subjects into a continuum of risk classes using quantiles as cut-off points to investigate how the selected SNPs and their predictive power were affected by the extreme subject selection strategy [1]. Such a selection strategy has previously been shown to increase statistical power in single-locus association analyses [26-29], whereas here we combined this strategy into a predictive modeling framework to detect panels of SNPs which do not necessary pass the level of statistical significance but can still classify subjects with an increasing degree of risk of developing atherosclerosis. Our stratified sampling procedure results in balanced low- and high-risk subject classes, provided that there are no ties in the outcome variables. Since the IMT recordings were made on three digits resolution, some of the subjects had exactly the same IMT value. In the 2001 follow-up study, for instance, there were only 171 unique IMT values among the 1,027 subjects. Such tie cases at the quantile levels were dealt with by including all the subjects with the quantile IMT-value, such as 15% or 85%, into the low risk or high risk class, respectively, even if this reduced the prediction accuracies to some extent (Figure 4). The categorical nature of the IMT levels makes the classification models more appropriate for the IMT prediction than the standard regression models. It is likely that novel and more efficient continuous modeling frameworks for the categorical SNP and IMT data need to be developed before moving toward the ‘apogee’ of the quantitative trait thinking for atherosclerosis [1].

Although prediction algorithms, such as the naïve Bayes can handle missing SNP data, the missing values can have an adverse effect on the overall performance of the predictive model. As our objective was to report reliable sets of SNPs that can predict the increasing IMT risk classes, we wanted to make sure that neither the prediction accuracies nor the SNPs reported were distorted because of the missing values, and therefore the subjects without SNP or IMT data were excluded in the current analysis. The IMT distributions were similar between the included and excluded subjects both in the 2001 and 2007 follow-up studies (Kolmogorov-Smirnov test D=0.034 and D=0.040, respectively, both with p>0.4). However, it is possible that some of the informative variants were filtered out during the initial selection phase in which the complete data matrix was constructed (see Figure S1). It is therefore expected that even better prediction accuracies, together with novel variants, will be obtained when the

same protocol will be applied to an unbiased genome-wide genotyping of SNPs in the same individuals. The possibility that many relevant genetic variants were not among the set of the 108 candidate SNPs used in the prediction studies here, may also partly explain the rather limited overlap between the SNP sets that were found to be most predictive of the 2001 and 2007 IMT-levels, as well as of its progression from 2001 to 2007 (Tables 2-4). On the other hand, the relatively high variability in the most predictive variants across the various IMT risk classes, which was also observed within each individual follow-up study (Table S1), is likely to reflect the genotype-specificity of the quantitative IMT phenotype.

Clinical significance and conclusion

Highly predictive genetic profiles could offer opportunities for many clinical and public health applications, ranging from guiding population-based screening procedures to the guidance of clinical decision making in terms of diagnostic or prognostic tests [30]. For instance, the finding that the genetic variants most predictive of sub-clinical atherosclerosis risk are mostly different from those of clinically manifesting CHD outcomes likely reflects the genetic heterogeneity of the disease pathogenesis and suggests that multiple panels of genetic markers may be needed to characterize its different development stages. While assessing that the genetic markers are predictive of the early disease risk is an essential first step in translating genetic profiles into medical and public health applications, it is still far from the eventual assessment of the net benefit of prevention strategies guided by genetic profiles. Once the reproducibility of the candidate markers has also been confirmed on other similar materials, one may consider proceeding to the next phases of biomarker development, involving, for instance, more targeted clinical immunoassays and evaluation studies in large and well-controlled study populations.

In conclusion, we have demonstrated, for the first time in a population-based follow-up study, that genetic variants, which do not necessarily meet the level of statistical significance, can contain added information according to which it is possible to classify subjects with different degrees of risk of developing atherosclerosis. The predictive modeling framework facilitates the usability of genetic information by discovering informative panels of variants, along with conventional risk factors, which may prove to have clinical utility in the early detection and management of sub-clinical atherosclerosis and other quantitative disorders.

References

1. Plomin R, Haworth CM, Davis OS (2009) Common disorders are quantitative traits. Opinion. Nat Rev Genet 10: 872-878.
2. Donnelly P (2008) Progress and challenges in genome-wide association studies in humans. Commentary. Nature 456: 728-731.
3. Maher B (2008) Personal genomes: The case of the missing heritability. News Feature. Nature 456: 18-21.
4. Humphries SE, Cooper JA, Talmud PJ, Miller GJ (2007) Candidate gene genotypes, along with conventional risk factor assessment, improve estimation of coronary heart disease risk in healthy UK men. Clin Chem 53: 8-16.
5. Morrison AC, Bare LA, Chambless LE, Ellis SG, Malloy M, et al. (2007) Prediction of coronary heart disease risk using a genetic risk score: the Atherosclerosis Risk in Communities Study. Am J Epidemiol 166: 28-35.
6. van der Net JB, Janssens AC, Defesche JC, Kastelein JJ, Sijbrands EJ, et al. (2009) Usefulness of genetic polymorphisms and conventional risk factors to predict coronary heart disease in patients with familial hypercholesterolemia. Am J Cardiol 103: 375-380.
7. van der Net JB, Janssens AC, Sijbrands EJ, Steyerberg EW (2009) Value of genetic profiling for the prediction of coronary heart disease. Am Heart J 158: 105-110.
8. Ioannidis JP (2009) Prediction of cardiovascular disease outcomes and established cardiovascular risk factors by genome-wide association markers. Circ Cardiovasc Genet 2: 7-15.
9. Paynter NP, Chasman DI, Paré G, Buring JE, Cook NR, et al. (2010) Association between a literature-based genetic risk score and cardiovascular events in women. JAMA 303: 631-637.

10. Samani NJ, Raitakari OT, Sipilä K, Tobin MD, Schunkert H, et al. (2008) Coronary artery disease-associated locus on chromosome 9p21 and early markers of atherosclerosis. Arterioscler Thromb Vasc Biol 28: 1679-1683.

11. Fan YM, Raitakari OT, Kähönen M, Hutri-Kähönen N, Juonala M, et al. (2009) Hepatic lipase promoter C-480T polymorphism is associated with serum lipids levels, but not subclinical atherosclerosis: The Cardiovascular Risk in Young Finns Study. Clin Genet 76: 46-53.

12. Simon R, Radmacher MD, Dobbin K, McShane LM (2003) Pitfalls in the use of DNA microarray data for diagnostic and prognostic classification. J Natl Cancer Inst 95: 14-18.

13. Frazer KA, Murray SS, Schork NJ, Topol EJ (2009) Human genetic variation and its contribution to complex traits. Nat Rev Genet 10: 241-251.

14. Moore JH, Williams SM (2009) Epistasis and its implications for personal genetics. Am J Hum Genet 85: 309-320.

15. Moore JH, Asselbergs FW, Williams SM (2010) Bioinformatics challenges for genome-wide association studies. Bioinformatics 26: 445-455.

16. Cordell HJ (2009) Genome-wide association studies: Detecting gene-gene interactions that underlie human diseases. Nat Rev Genet 10: 392-404.

17. Phillips PC (2008) Epistasis: the essential role of gene interactions in the structure and evolution of genetic systems. Review. Nat Rev Genet 9: 855-867.

18. Clayton DG (2009) Prediction and interaction in complex disease genetics: experience in type 1 diabetes. PLoS Genet 5: e1000540.

19. Kristiansson K, Ilveskoski E, Lehtimäki T, Peltonen L, Perola M, et al. (2008) Association analysis of allelic variants of USF1 in coronary atherosclerosis. Arterioscler Thromb Vasc Biol 28: 983-989.

20. The Wellcome Trust Case Control Consortium (2007) Genome-wide association study of 14 000 cases of seven common diseases and 3 000 shared control. Nature 447: 661-678.

21. Kathiresan S, Melander O, Guiducci C, Surti A, Burtt NP, et al. (2008) Six new loci associated with blood low-density lipoprotein cholesterol, high-density lipoprotein cholesterol or triglycerides in humans. Nat Genet 40:189-197.

22. Bevan S, Lorenz MW, Sitzer M, Markus HS (2009) Genetic variation in the leukotriene pathway and carotid intima-media thickness: a 2-stage replication study. Stroke 40: 696-701.

23. Barrett JC, Clayton DG, Concannon P, Akolkar B, Cooper JD, et al. (2009) Genome-wide association study and meta-analysis find that over 40 loci affect risk of type 1 diabetes. Nat Genet 41: 703 – 707.

24. Barrett JC, Hansoul S, Nicolae DL, Cho JH, Duerr RH, et al. (2008) Genome-wide association defines more than 30 distinct susceptibility loci for Crohn's disease. Nat Genet 40: 955-962.

25. Gregersen PK, Amos CI, Lee AT, Lu Y, Remmers EF, et al. (2009) REL, encoding a member of the NF-kappaB family of transcription factors, is a newly defined risk locus for rheumatoid arthritis. Nat Genet 41: 820-823.

26. Schork NJ, Nath SK, Fallin D, Chakravarti A (2000) Linkage disequilibrium analysis of biallelic DNA markers, human quantitative trait loci, and threshold-defined case and control subjects. Am J Hum Genet 67: 1208-1218.

27. Lanktree MB, Hegele RA, Schork NJ, Spence JD (2010) Extremes of unexplained variation as a phenotype: an efficient approach for genome-wide association studies of cardiovascular disease. Circ Cardiovasc Genet 3: 215-221.

28. Zhang G, Nebert DW, Chakraborty R, Jin L (2006) Statistical power of association using the extreme discordant phenotype design. Pharmacogenet Genomics 16: 401-143.

29. Eguchi T, Maruyama T, Ohno Y, Morii T, Hirao K, et al. (2009) Possible association of tumor necrosis factor receptor 2 gene polymorphism with severe hypertension using the extreme discordant phenotype design. Hypertens Res 32: 775-779.

30. Kraft P, Wacholder S, Cornelis MC, Hu FB, Hayes RB, et al. (2009) Beyond odds ratios: communicating disease risk based on genetic profiles. Perspective. Nat Rev Genet 10: 264-9.
